# The Diagnosis and Treatment of Venereal Diseases in General Practice

**Published:** 1919-12

**Authors:** 


**The Diagnosis and Treatment of Venereal Diseases in General Practice.** By L. W. Harrison, D.S.O., Lieut.-Col., R.A.M.C. Pp. xvii., 482. London: Oxford Medical Publications. 1918. Price 21s. net.

Now that the subject of venereal disease has attracted more general interest among the lay people than ever before, and now that the suppression of unqualified practice in venereal disease has obtained a more stringent legal sanction, it is reasonable for the public to look to the general practitioner for a thorough knowledge of the diagnosis and treatment of these diseases.

In order to assist the general practitioner in obtaining this necessary knowledge, Lieut.-Col. Harrison has written this text-book from a wealth of clinical material and experience, and it may justly be considered as the standard text-book on venereal diseases in English.

While concise, it omits nothing of importance, yet it brings the student up to date in modern diagnosis and the technique of treatment.

The use and abuse of Salvarsan and its substitutes receive ample notice, as should be the case in a modern text-book on the subject, and the position and reliability of the Wassermann test is discussed with judgment and experience.

The book is admirably compiled and arranged, while the illustrations are excellent and well reproduced.

By its arrangement in paragraphs the book is easy of reference, and a good index helps materially to this end.

The author is recognised as the foremost authority in the treatment of venereal diseases in this country, and he is very much to be congratulated in that he has managed to produce so admirable a work during the war.

